# Molecular Epidemiology of HIV-1 Infection among Men who Have Sex with Men in Taiwan in 2012

**DOI:** 10.1371/journal.pone.0128266

**Published:** 2015-06-03

**Authors:** Szu-Wei Huang, Sheng-Fan Wang, Ángel E. Cowó, Marcelo Chen, Yu-Ting Lin, Chun-Po Hung, Yi-Hsien Chen, Jyh-Yuan Yang, Hung-Jen Tang, Yi-Ming Arthur Chen

**Affiliations:** 1 Institute of Microbiology and Immunology, National Yang Ming University, Taipei 11221, Taiwan; 2 Center for Infectious Disease and Cancer Research, Kaohsiung Medical University, Kaohsiung 80708, Taiwan; 3 Department of Medical Laboratory Science and Biotechnology, Kaohsiung Medical University, Kaohsiung 80708, Taiwan; 4 International Health Program, National Yang Ming University, Taipei 11221, Taiwan; 5 Department of Urology, Mackay Memorial Hospital, Taipei 10449, Taiwan; 6 Department of Cosmetic Applications and Management, Mackay Junior College of Medicine, Nursing and Management, Taipei 25245, Taiwan; 7 School of Medicine, Mackay Medical College, New Taipei City 25245, Taiwan; 8 Rainbow Queer Health and Culture Center, Living with Hope Organization, Taiwan Society of Preventive Medicine, Taipei 10084, Taiwan; 9 Centers for Disease Control, Taipei 10050, Taiwan; 10 Department of Medicine, Chi Mei Medical Center, Tainan 71067, Taiwan; 11 Department of Health and Nutrition, Chia Nan University of Pharmacy and Science, Tainan 71710, Taiwan; 12 Department of Microbiology and Immunology, Institute of Medical Research and Institute of Clinical Medicine, College of Medicine, Kaohsiung Medical University, Kaohsiung 80708, Taiwan; UNSW Australia, AUSTRALIA

## Abstract

The number of men who have sex with men (MSM) infected with HIV-1 in Taiwan has increased rapidly in the past few years. The goal of this study was to conduct a molecular epidemiological study of HIV-1 infection among MSM in Taiwan to identify risk factors for intervention. Voluntary counseling program and anonymous testing were provided to patrons at 1 gay bar, 7 night clubs and 3 gay saunas in Taipei and New Taipei Cities in 2012. HIV-1 subtypes were determined using gag subtype-specific PCR and phylogenetic analysis by env sequences. Recent HIV-1 infection was determined using LAg-Avidity EIA. In-depth interviews and questionnaires were used to identify risk factors. The prevalence and incidence of HIV-1 among MSM in Taiwan were 4.38% (53/1,208) and 3.29 per 100 person-years, respectively. Of 49 cases genotyped, 48 (97.9%) were infected with subtype B and 1 with CRF01_AE (2%). Phylogenetic analysis of 46 HIV-1 strains showed that 25 (54.4%) subtype B strains formed 9 clusters with each other or with other local strains. The CRF01_AE case clustered with a reference strain from a Thai blood donor with bootstrap value of 99. Multivariate logistic regression analysis showed that risk factors associated with HIV-1 infection included use of oil-based solution as lubricant (vs. saliva or water-based lubricants, OR= 4.23; p <0.001); exclusively receptive role (vs. insertive role, OR= 9.69; p <0.001); versatile role (vs. insertive role, OR= 6.45; p= 0.003); oral sex (vs. insertive role, OR= 11.93; p= 0.044); times of sexual contact per week (2-3 vs. zero per week, OR= 3.41; p= 0.021); illegal drug use (OR= 4.12; p <0.001); and history of sexually transmitted diseases (OR= 3.65; p= 0.002). In conclusion, there was no new HIV-1 subtype or circulating recombinant form responsible for the increase of HIV-1 among MSM in Taiwan in 2012. Misuse of oil-based solution as lubricant is a new risk factor identified among MSM in Taiwan. The Taiwan’s Centers for Disease Control has created a video (www.youtube.com/watch?v=BinExvvOTMM&feature=iv&src_vid=BW81-PfmY3E&annotation_id=annotation_2436493705) to correct such misconception in its AIDS prevention campaign.

## Introduction

According to the Joint United Nations Program on HIV/AIDS (UNAIDS) report, 35.3 million people worldwide were living with HIV in 2012 [1]. A recent review has shown that the global trend of HIV-1 infection among men who have sex with men (MSM) has continued to increase, especially in East Asia, Africa, and Russia [2]. By the end of 2012, there were 25,081 cases of HIV infection been reported to Taiwan’s Centers for Disease Control (CDC) and Taiwanese nationals accounted for 96.6% of those cases (Taiwan CDC report 2012; http://www.cdc.gov.tw/info.aspx?treeid=1f07e8862ba550cf&nowtreeid=6c5ea6d932836f74&tid=58114AFB86117F53). Among them, homosexual men accounted for 41.9% of cases, followed by injection drug users (IDUs, 27.6%), heterosexuals (20.3%) and bisexual men (8.3%). There was an explosive outbreak of circulating recombinant form (CRF) _07BC among Taiwanese IDUs from 2004 to 2006 [3–5]. However, the HIV epidemic took a turn in 2007 as HIV-1 infection among IDUs decreased rapidly due to the implementation of harm reduction programs [6] and infection among MSM kept increasing exponentially [7]. Therefore, special attention needs to be given to any new HIV-1 subtype or CRF transmitted to a Taiwanese MSM. However, studies of the HIV-1 subtypes in MSM in Taiwan after 2009 are lacking.

Risk factors for HIV infection in MSM include unprotected anal intercourse [8–11], illegal drug use [11–13], a history of other sexually transmitted diseases (STDs) [12], condom use, multiple sex partners and role during anal intercourse [8, 11, 14, 15]. Previous studies have shown an association between high level of substance use, sexual risk behavior and risk of HIV infection in young MSM [16–19]. A study by Hikada et al. reported that 45% of MSM ever used one type of drug and 19.6% ever used more than one type of drugs [20]. According to the San Francisco Young Men’s Study, 43% of the subjects had used multiple illegal drugs [21].

The objective of this report was to conduct a molecular epidemiological study of HIV-1 subtypes and CRFs among MSM in Taiwan in 2012. Viral subtypes and recombinants and information on participants’ behavioral characteristics were obtained in order to investigate possible associations between subtypes and potential risk factors for HIV infection among MSM in Taiwan.

## Materials and Methods

### Subjects

Before beginning the study, we contacted commercial gay venues (one gay bar, seven gay night clubs and three gay saunas) in Taipei City to inquire about their willingness to provide space for our study. Anonymous, self-completed questionnaires and blood samples were collected from patrons at these venues. The information asked in our questionnaires includes demographics, HIV testing history, illegal drug use and sexual risk behavior (questionnaire available online at http://cicar.kmu.edu.tw/index.php/zh-TW/). All study participants received pre-test counseling. All study participants also received post-test counseling when results were given. Those who were HIV antibody negative received information on HIV prevention and were encouraged to undergo follow-up every 3 months. Those found to be HIV antibody positive were provided with information on where to obtain clinical assessment and treatment. This study was conducted with the approval of the institutional review board of the Mackay Memorial Hospital, Taipei, Taiwan. Written informed consent was obtained from participants before the interview and testing.

### Serologic tests for HIV-1, syphilis and recent HIV-1 infections

HIV diagnosis was determined with a recombinant HIV enzyme immunoassay (Murex Diagnostics Limited, Dartford, UK). Positive samples were confirmed by HIV Western blot 2.2 (Genelab Technologies, Inc., Singapore). Active syphilis infection was defined using the following criteria: seropositive in Treponema pallidum hemagglutination assay (FTI-SERODIA-TPPA, Fujirebio Taiwan Inc., Taoyuan, Taiwan) and antibody titer ≥ 160 in rapid plasma regain (Macro-Vue RPR Kits, Becton, Dickinson and Company, NJ, USA) [22]. Recent HIV-1 infection was determined using LAg-Avidity EIA (Sedia Biosciences Corporation Portland, OR, USA). This assay was performed according to the manufacturer’s instructions. Briefly, recent infections were determined by a calibrator-normalized OD value (ODn) below the cut off value of 1. The mean duration of recency (MDR) was 141 days according to the manufacturer’s description. The annual HIV incidence was calculated using a formula provided by the manufacturer [23].

### HIV-1 subtyping and phylogenetic analysis

DNA was extracted from peripheral blood mononuclear cells (PBMC) using QIAmp blood extraction kits (QIAgen, Valencia, CA.). HIV-1 *gag* and *env* regions were used to determine the subtypes by nested multiplex polymerase chain reaction (PCR) and phylogenetic analysis, respectively. A gag subtype-specific PCR with specific primers modified from a method previously described by Wei, et al. was used to determine HIV-1 viral subtypes and/or recombinants [24]. Extracted DNA was amplified by nested PCR using the TaKaRa Ex Taq system (Takara Bio Inc, Japan) according to the manufacturer’s instructions. HIV-1 *env* (C2-V5) was obtained through direct sequencing of the PCR products using a DNA analyzer (ABI 3730, Applied Biosystems, Foster City, CA). The nucleotide sequences of the patients were aligned with different reference sequences from Los Alamos HIV Database (http://www.hiv.lanl.gov) by BioEdit [25]. Phylogenetic trees were constructed using the best fit nucleotide substitution model [26] and the Neighbor-joining (NJ) and maximum likelihood (ML) methods using MEGA 6.06 [27] Phylip 3.69 [28]. The substitution model TN93 + G model was used to calculate the evolutionary distance and was followed by bootstrap value analysis with 1000 replicates for NJ tree construction [29]. To determine whether or not the HIV-1 infection was associated with foreign countries, sequences representing isolates from different regions in the world, in particular Asian countries and local sequences from Taiwan were used as reference sequences in the analysis. The local sequences were from subjects who were MSM (15/34), heterosexual (16/34) or IDUs (3/34) collected in our lab in 2004–2012. The selection of a bootstrap value of 70% was based on other references as well as on our past experiences. According to a study reported by Hills et al., the selection of bootstrap value of 70 correlates to a 95% probability of a cluster being real [30]. Therefore, bootstrap values >70 indicated significance of the sequences clustered.

### Statistical analysis

Pearson's χ2 test and Fisher’s exact test were performed in a univariate analysis to investigate differences in demographic data and consumer behavior patterns among MSM. A multivariate logistic regression analysis was performed to identify risk factors associated with HIV-1 infection. The multivariate analyses were performed using SAS version 9.4 (SAS Institute, Inc., Gary, NC).

### Nucleotide sequence accession numbers

The HIV-1 env sequences of 34 local control cases and 46 MSM obtained in this study were deposited in GenBank with accession numbers KM668624—KM668657 and KM668658—KM668703, respectively. The 34 HIV-1 env sequences were identified in our previous studies of MSM, IDUs and heterosexuals since we used them as local controls in our phylogenetic tree analysis.

## Results

### Demographic characteristics

A total of 1,208 participants were recruited in this study. The mean age of participants was 28.6 years (range, 18–57 years). Most of the participants were single (94%), self-identified homosexuals (80.2%) and with educational levels ranging from college (66.1%) to graduate school (16.4%). Over half of the participants (54.5%) were office workers and 20.9% were students. Most of the participants were recruited from gay night clubs (53.6%) and gay bar (33.9%), and the remaining 12.6% from gay saunas (Table 1).

**Table 1:** Demographic data of patrons from different gay venues participated in this study.

	HIV-1 (+)	HIV-1 (-)	Total	
Variable	N = 53	N = 1,155	N = 1,208	p
	n (%)	n (%)	n (%)	
Age				0.970§
18–29	27 (50.9)	568 (49.2)	595 (49.3)	
30–39	12 (22.6)	289 (25.0)	301 (24.9)	
40–49	2 (3.8)	58 (5.0)	60 (4.9)	
≥ 50	0 (0)	4 (0.3)	4 (0.3)	
NA	12 (22.6)	236 (20.4)	248 (20.5)	
Mean ± SD	28.1±5.0	28.6±6.5	28.6±6.4	0.625†
Marital status				0.162§
Single	47 (88.7)	1,089 (94.3)	1136 (94.0)	
Married	1 (1.9)	15 (1.3)	16 (1.3)	
Divorced/Separated/Widowed	1 (1.9)	12 (1.1)	13 (1.1)	
NA	4 (7.5)	39 (3.4)	43 (3.6)	
Sexual orientation				0.331§
Heterosexual	0 (0)	32 (2.8)	32 (2.6)	
Homosexual	49 (92.5)	920 (79.7)	969 (80.2)	
Bisexual	4 (7.5)	178 (15.4)	182 (15.1)	
NA	0 (0)	21 (1.8)	21 (1.7)	
Education				0.555*
≤ Junior high school	1 (1.9)	21 (1.8)	22 (1.8)	
Senior high school	8 (15.1)	162 (14.0)	170 (14.1)	
College	39 (73.6)	760 (65.8)	799 (66.1)	
≥ Graduate	5 (9.43)	193 (16.7)	198 (16.4)	
NA	0 (0)	19 (1.6)	19 (1.6)	
Occupation				0.143§
Student	6 (11.3)	247 (21.4)	253 (20.9)	
Government employees	7 (13.2)	73 (6.3)	80 (6.6)	
Office worker	33 (62.3)	625 (54.1)	658 (54.5)	
Professional	2 (3.8)	92 (8.0)	94 (7.8)	
Unemployed/Other	5 (9.4)	97 (8.4)	102 (8.4)	
NA	0 (0)	21 (1.8)	21 (1.7)	
Place				0.763*
Gay bar (1)	16 (30.2)	393 (34.0)	409 (33.9)	
Gay night clubs (7)	31 (58.5)	616 (53.3)	647 (53.6)	
Gay saunas (3)	6 (11.3)	146 (12.6)	152 (12.6)	

*, Chi-square test.

^§^, Fisher exact test.

^†^, Student T Test.

### Prevalence and incidence rates of HIV and syphilis among MSM

Fifty-three participants were diagnosed with HIV‐1 infection in 2012. The overall HIV‐1 prevalence was 4.38%. The results of LAg-Avidity EIA showed that 16 of the 53 HIV-1 infected MSM were recent seroconverters and the resultant incidence was 3.29 per 100 person-years (Table 2). The prevalence and incidence were not statistically different between gay venues (p = 0.763 and 0.496 respectively). The prevalence of syphilis among MSM in our cohort was 2.15%.

**Table 2:** Prevalence and incidence rates of HIV-1 infection among MSM from different gay venues in Taiwan.

	No. of participants screened	No. of HIV-1 positive (%)	No. of recent seroconverters^a^	Incidence rate (per 100 person-years)
Gay bar	409	16 (3.91)	4	2.36
Gay night clubs	647	31 (4.79)	9	3.46
Gay saunas	152	6 (3.94)	3	5.14
Total	1,208	53 (4.38)	16	3.29

^a^, Determined by LAg-Avidity EIA.

### Distribution of HIV-1 subtypes among MSM

HIV-1 subtypes were determined by gag subtype-specific PCR amplification, and confirmed by sequencing and phylogenetic analysis of the env gene. During the study period, all HIV‐1 positive samples were available for genotyping, and 92.5% (49/53) were successfully genotyped. In 4 of our patients the subtypes could not be determined due to the limited quantity of the specimens. Therefore, their subtypes were classified as indeterminate. The result of gag subtype-specific PCR showed that 97.9% (48/49) of patients were infected with subtype B and one patient from gay sauna was infected with CRF01_AE. Forty-six sequences were amplified successfully by env-specific PCR. The NJ distance method classified 97.8% (45/46) of patients as infected with HIV-1 subtype B. One HIV-1 patient was classified as CRF01_AE. In addition, three HIV-1 subtype B infected patients were genotyped successfully by gag subtype-specific PCR but not by env-specific PCR. However, the results of HIV-1 subtype genotyping showed a consensus between gag subtype-specific PCR and phylogenetic analysis of env region (Table 3).

**Table 3:** HIV-1 subtypes identified among MSM in Taiwan.

	B	CRF01_AE	Indeterminate	Total
	n (%)	n (%)	n (%)	N (%)
Gay bar	14 (87.5)	0 (0)	2 (12.5)	16 (100)
Gay night clubs	29 (93.5)	0 (0)	2 (6.5)	31 (100)
Gay saunas	5 (83.3)	1 (16.7)	0 (0)	6 (100)
Total	48 (90.6)	1 (1.9)	4 (7.5)	53 (100)

Taken together, except for a patient from gay sauna who was infected with CRF01_AE, all other patients (97.9%, 48/49) were infected with HIV-1 subtype B.

### The origin and dissemination pathways of HIV-1 subtypes among MSM

**Fig 1:**
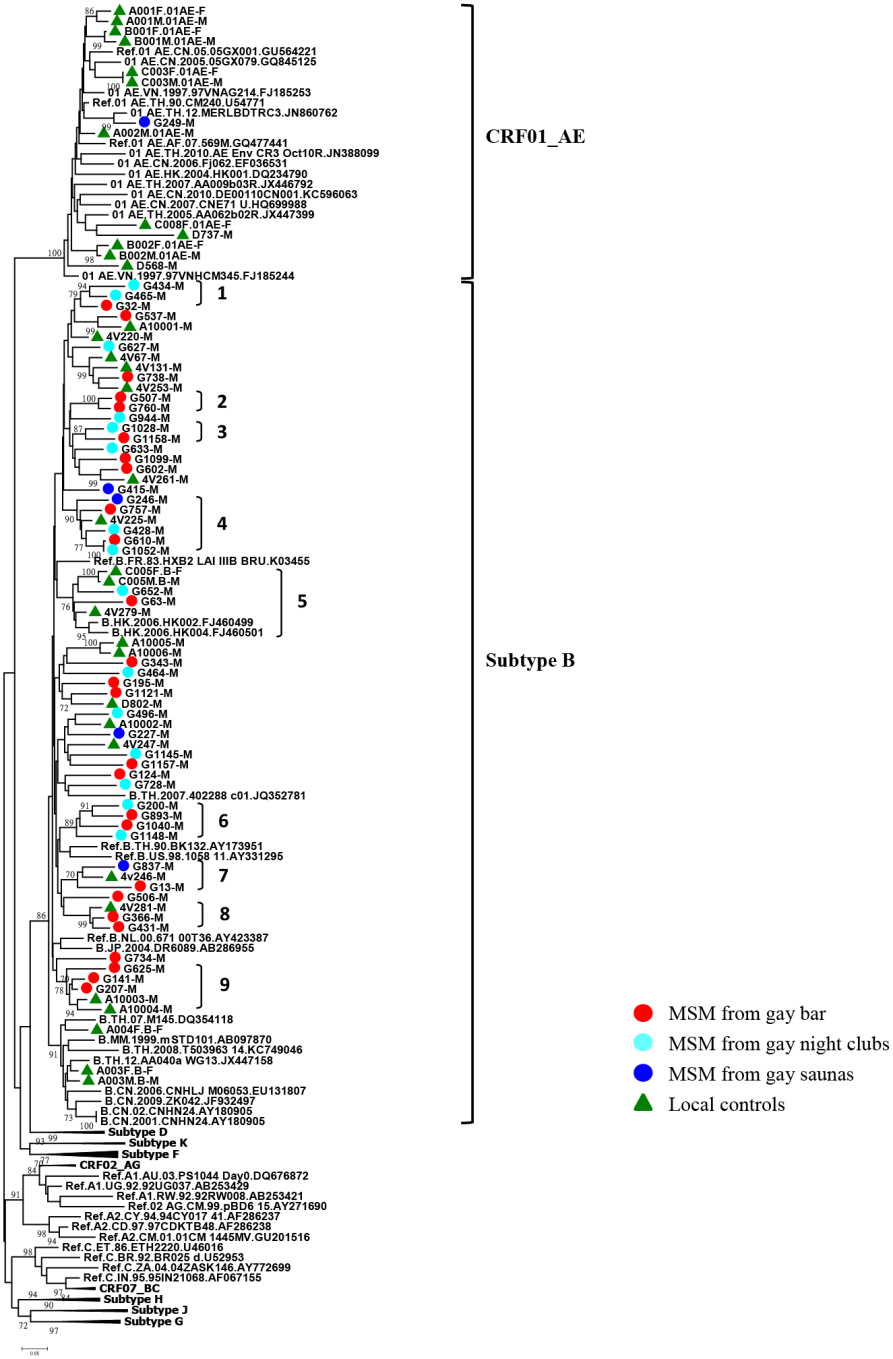
Phylogenetic analysis of HIV-1 strains identified from MSM in Taiwan in 2012. Neighbor-Joining tree based on *env* (7077–7619 nucleotide residues of HXB2) nucleotide sequences. All branch lengths are drawn to scale. Red circle indicates MSM patients from a gay bar. Aqua circle indicates MSM patients from gay night clubs. Blue circle indicates MSM patients from gay saunas. Green triangle indicates local controls from Taiwan. Clusters with bootstrap value ≥ 70 were labeled from numbers 1 to number 9.

Phylogenetic analysis using *env V3-V5 loop* gene was conducted to elucidate the transmission relationship between Asian countries and Taiwanese reference sequences (local control sequences). Consistent tree topologies were observed in both the NJ and ML trees. Of these local controls, 22 patients were infected with subtype B and 12 patients were infected with CRF01_AE. The results showed that most subtype B strains from gay venues clustered among themselves or with local controls recruited from 2004 to 2012 from different regions of Taiwan (13 clusters with bootstrap value > 70). Only one of the 46 HIV-1 patients from gay saunas clustered in clade CRF01_AE (G249). This sequence significantly clustered with a reference strain from Thailand (accession number: JN860762) with a bootstrap value of 99%. G249 was a patron recruited from a gay sauna and reported himself as a homosexual (Fig 1). Therefore, we conclude that HIV-1 subtype B strains from gay bar, gay night club and gay sauna patrons were of local origin and no non-B subtypes circulated in the MSM population.

**Fig 2:**
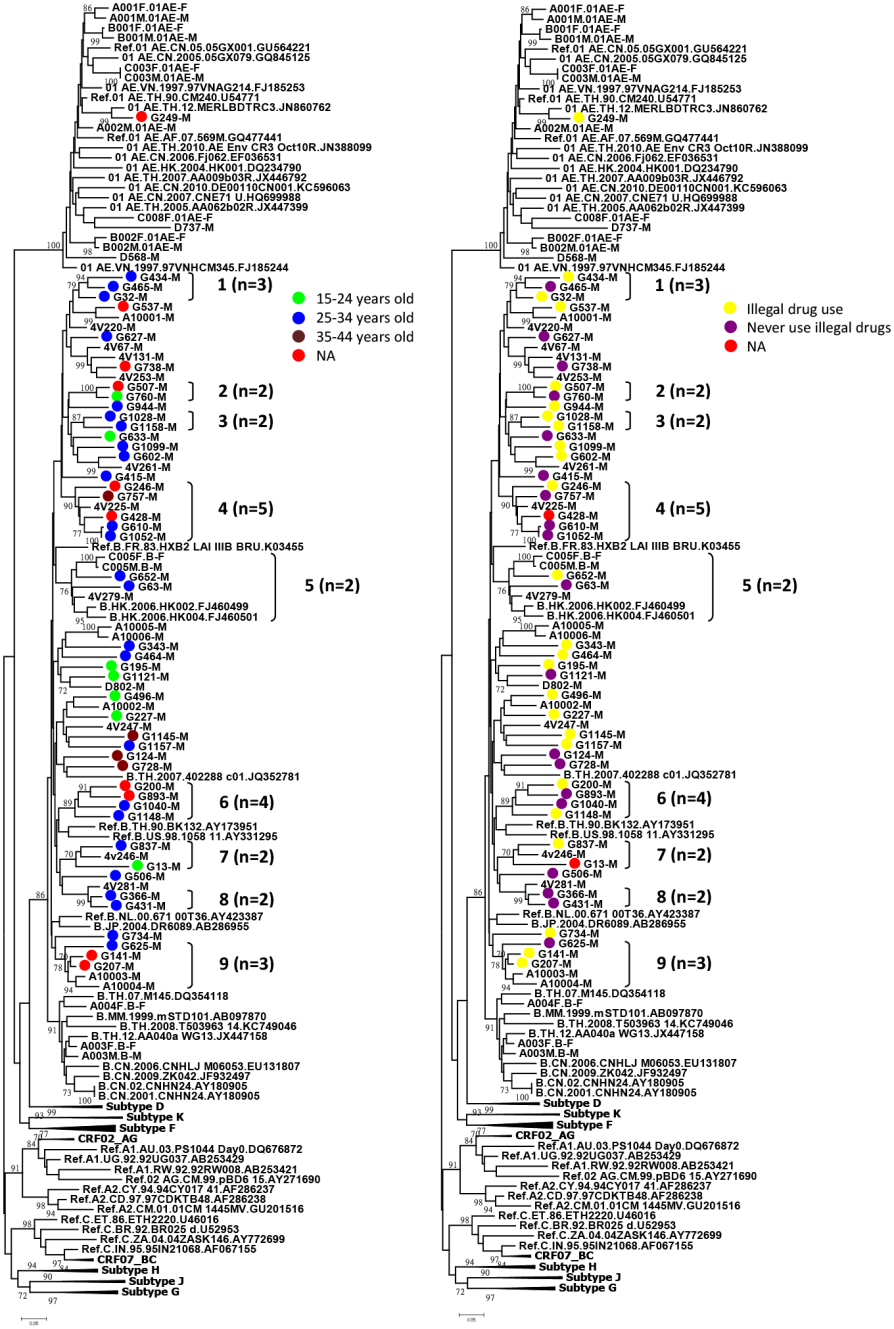
Analyses of the characteristics of the clusters of the phylogenetic trees based on age or illegal drug use. (A) Characteristics of the cluster based on age. Green circle indicates 15–24 years old. Blue circle indicates 25–34 years old. Brown circle indicates 35–44 years old. Red circle indicates data not available (NA). (B) Characteristics of the cluster based on illegal drug use. Yellow circle indicates illegal drug use. Purple circle indicates no illegal drug use. Red circle indicates data not available (NA). Clusters with bootstrap value ≥ 70 were labeled from numbers 1 to number 9 and number of individuals per cluster recruited in this study.

The characteristics of clusters of MSM infected with HIV-1 are shown in Figs 1 and 2. Twenty-five subtype B strains formed 9 clusters with each other or with other local controls. When we analyzed the clusters by age and illegal drug use, the results showed that there was one cluster (No. 3) of two members, both of whom were young (between 25–34 years old) who used illegal drugs (Fig 2). In addition, there were two clusters (No. 1 and No.5) of young MSM, although not all reported illegal drug use. The rest of the clusters were composed of either young MSM with or without reported use of illegal drugs (No. 8) or included MSM of a wide age range with or without reported use of illegal drugs (No. 4, 7) (S1 Table).

### Risk factors associated with HIV-1 infection among MSM

Univariate and multivariate risk factor analyses for HIV-1 infection are presented in Tables 4 and 5. According to univariate analysis, exclusively receptive role and versatile role (vs. exclusively insertive role, OR = 6.79 and 4.92; p = 0.003 and 0.009), times of sexual contact per week (2–3 vs. zero per week, OR = 3.34; p = 0.015), illegal drug use (vs. no use, OR = 4.28; p <0.001), use of 3 or more drugs (vs. single drug use, OR = 5.37; p = 0.002), use of oil-based lubricants during sexual intercourse (vs. saliva or water-based lubricants, OR = 3.18; p = 0.004), and history of STDs (vs. no history, OR = 4.24; p <0.001) were all associated with an increased risk of HIV-1 infection. Multivariate analysis was conducted of the risk factors mentioned above. The risk factors significantly associated with HIV-1 infection were exclusively receptive role, versatile role and oral sex (vs. exclusively insertive role, OR = 9.69, 6.45 and 11.93; p <0.001, 0.003 and 0.044, respectively), times of sexual contact per week (2–3 vs. zero per week, OR = 3.41; p = 0.021), illegal drug use (vs. no use, OR = 4.12; p <0.001), use of oil-based lubricants during sexual intercourse (vs. saliva or water-based lubricants, OR = 4.23; p <0.001) and history of STDs (vs. no history, OR = 3.65; p = 0.002) (Table 5).

**Table 4:** Univariate analysis of risk factors for HIV-1 infection among MSM in Taiwan.

	HIV-1 (+)	HIV-1 (-)		
Variable	N = 53	N = 1,155	Odds ratio*	p
	n (%)	n (%)		
Pay for condom				
Yes	47 (88.7)	934 (80.9)	1	1
No	5 (9.4)	189 (16.4)	0.53	0.178
NA	1 (1.9)	32 (2.8)	0.62	0.643
Knowledge of PEP				
Yes	13 (24.5)	224 (19.4)	1	1
No	35 (66.0)	782 (67.7)	0.31	0.436
NA	5 (9.43)	149 (12.9)	0.58	0.307
Number of sexual partners				
0	7 (13.2)	165 (14.3)	1	1
1	18 (33.9)	523 (45.3)	0.81	0.645
2–3	14 (26.4)	260 (22.5)	1.27	0.615
≥ 4	2 (3.8)	55 (4.8)	0.86	0.850
NA	12 (22.6)	152 (13.2)	1.86	0.204
Role during anal intercourse				
Exclusively insertive	3 (5.7)	265 (22.9)	1	1
Exclusively receptive	16 (30.2)	208 (18.0)	6.79	0.003
Versatile	32 (60.4)	575 (49.8)	4.92	0.009
Oral sex	1 (1.9)	19 (1.6)	4.65	0.192
NA	1 (2.9)	88 (7.6)	1.00	0.997
Times of sexual contact per week				
0	6 (11.3)	218 (18.9)	1	1
1	23 (43.4)	527 (45.6)	1.59	0.322
2–3	15 (28.3)	163 (14.1)	3.34	0.015
≥ 4	1 (1.9)	58 (5.0)	0.63	0.668
NA	8 (15.1)	189 (16.4)	1.54	0.433
Illegal drug use				
No	21 (39.6)	742 (64.2)	1	1
Yes	28 (52.8)	231 (20.0)	4.28	<0.001
NA	4 (7.5)	182 (15.8)	0.78	0.647
Types or numbers of illegal drugs use				
Single drug	8 (15.1)	124 (10.7)	1	1
Ketamine + MDMA^a^	10 (18.9)	61 (5.3)	2.54	0.062
Ketamine + Viagra	1 (1.9)	11 (0.9)	1.41	0.757
Ketamine + Nimetazepam	0 (0.0)	1 (0.1)	<0.001	0.996
3 or more drugs	9 (16.9)	26 (2.3)	5.37	0.002
Others	0 (0.0)	8 (0.7)	<0.001	0.989
Never use drugs	21 (39.6)	742 (64.2)	0.44	0.053
NA	4 (7.5)	182 (15.8)	0.34	0.084
Sexual contact after illegal drug use (n = 28)		(n = 231)		
Never	5 (17.9)	44 (19.1)	1	1
Rarely/Occasionally/ Frequently/Always	17 (60.7)	107 (46.3)	1.39	0.534
Condom use after illegal drug use (n = 28)		(n = 231)		
Never	4 (14.3)	21 (9.1)	1	1
Rarely/Occasionally/ Frequently/Always	22 (78.6)	173 (74.9)	0.67	0.494
Oil- based lubricants during sexual intercourse				
Saliva or water- based	9 (16.9)	343 (29.7)	1	1
Oil- based^b^	25 (47.2)	300 (25.9)	3.18	0.004
NA	19 (35.9)	512 (44.3)	1.41	0.399
History of sexually transmitted diseases				
No	24 (45.3)	737 (63.8)	1	1
Yes	12 (22.6)	87 (7.5)	4.24	<0.001
NA	17 (32.1)	331 (28.7)	1.58	0.159
Frequency of condom use				
Always	20 (37.7)	484 (41.9)	1	1
Frequently/Occasionally/ Rarely/Never	30 (56.6)	511 (44.2)	1.42	0.235
NA	3 (5.7)	160 (13.9)	0.45	0.207
Frequency of lubricant use				
Always	42 (79.3)	805 (69.7)	1	1
Frequently/Occasionally/ Rarely/Never	10 (18.9)	224 (19.4)	0.86	0.665
NA	1 (1.9)	126 (10.9)	0.15	0.064

*, Chi-square test;

^a^, MDMA = 3,4-methylenedioxy-N-methylamphetamine or so called ecstasy

^b^, Soap, baby oil, Vaseline and petroleum jelly

**Table 5:** Multivariate logistic regression of risk factors for HIV-1 infection among MSM in Taiwan.

	HIV-1 (+)	HIV-1 (-)		
Variable	N = 53	N = 1,155	Odds ratio*	p
	n (%)	n (%)		
Occupation				
Student	6 (11.3)	247 (21.4)	1	1
Government employees	7 (13.2)	73 (6.3)	2.99	0.070
Office worker	33 (62.3)	625 (54.1)	2.28	0.083
Professional	2 (3.8)	92 (8.0)	1.25	0.795
Unemployed/Other	5 (9.4)	97 (8.4)	2.22	0.225
NA	0 (0)	21 (1.8)	<0.001	0.986
Role during anal intercourse				
Exclusively insertive	3 (5.7)	265 (22.9)	1	1
Exclusively receptive	16 (30.2)	208 (18.0)	9.69	<0.001
Versatile	32 (60.4)	575 (49.8)	6.45	0.003
Oral sex	1 (1.9)	19 (1.6)	11.93	0.044
NA	1 (2.9)	88 (7.6)	2.07	0.544
Times of sexual contact per week				
0	6 (11.3)	218 (18.9)	1	1
1	23 (43.4)	527 (45.6)	1.49	0.416
2–3	15 (28.3)	163 (14.1)	3.41	0.021
≥ 4	1 (1.9)	58 (5.0)	0.39	0.421
NA	8 (15.1)	189 (16.4)	2.33	0.151
Illegal drug use				
No	21 (39.6)	742 (64.2)	1	1
Yes	28 (52.8)	231 (20.0)	4.12	<0.001
NA	4 (7.5)	182 (15.8)	0.72	0.572
Oil-based lubricants during sexual intercourse				
Saliva or water- based	9 (16.9)	343 (29.7)	1	1
Oil-based ^a^	25 (47.2)	300 (25.9)	4.23	<0.001
NA	19 (35.9)	512 (44.3)	1.67	0.241
History of sexually transmitted diseases				
No	24 (45.3)	737 (63.8)	1	1
Yes	12 (22.6)	87 (7.5)	3.65	0.002
NA	17 (32.1)	331 (28.7)	1.75	0.109

*, Chi-square test

^a^, Soap, baby oil, Vaseline and petroleum jelly

### Illegal drug use among MSM

A total of 259 participants reported use of one or more types of illegal drugs. As shown in Table 6, the top three types of drugs abused by MSM were RUSH (Alkyl nitrites, 44.7%), MDMA (9.8%) and Ketamine (9.8%). HIV-1-infected MSM had significantly higher rate (17.9%, 5/28) of Ketamine, MDMA and Viagra use than HIV-1 negative MSM (5.2%, 12/231). The difference of the rates between these two groups was statistically significant (p = 0.011). It is worthy to note that there were two HIV-1-infected MSM reporting use of 6 drugs (Table 6).

**Table 6:** Distribution of different types or numbers of illegal drugs used among MSM in Taiwan.

	HIV-1 (+)	HIV-1 (-)	Total	
Variable	N = 28 (%)	N = 231 (%)	N = 259 (%)	p
	n (%)	n (%)	n (%)	
Single drug	n = 8	n = 124	n = 132	
RUSH	4 (50.0)	55 (44.4)	59 (44.7)	1§
MDMA	1 (12.5)	12 (9.7)	13 (9.8)	0.574§
Ketamine	1 (12.5)	12 (9.7)	13 (9.8)	0.574§
Marijuana	0 (0.0)	12 (9.7)	12 (9.1)	1§
LSD	0 (0.0)	4 (3.2)	4 (3.0)	1§
PCP	0 (0.0)	1 (0.8)	1(0.8)	1§
Others	2 (25.0)	28 (22.6)	30 (22.7)	1§
Two drugs	n = 11	n = 73	n = 84	
Ketamine + MDMA	10 (35.7)	61 (26.4)	71 (27.4)	0.297*
Ketamine + Viagra	1 (3.6)	11 (4.8)	12 (4.6)	1§
Ketamine + Nimetazepam	0 (0.0)	1 (0.4)	1 (0.4)	1§
≥ 3 drugs	n = 9	n = 26	n = 35	
Ketamine + MDMA + Viagra	5 (17.9)	12 (5.2)	17 (6.6)	0.011*
Ketamine + MDMA + Nimetazepam	1 (3.6)	1 (0.4)	2 (0.8)	0.205§
Ketamine + MDMA + Viagra + Marijuana	0 (0.0)	7 (3.0)	7 (2.7)	1§
Ketamine + MDMA + Viagra + Nimetazepam	0 (0.0)	2 (0.9)	2 (0.8)	1§
Ketamine + MDMA + Marijuana + Nimetazepam	0 (0.0)	1 (0.4)	1 (0.4)	1§
Ketamine + MDMA + Viagra + Marijuana + Nimetazepam	1 (3.6)	3 (1.3)	4 (1.5)	0.369§
Ketamine + PCP + MDMA + Viagra + Marijuana + Nimetazepam	1 (3.6)	0 (0.0)	1 (0.4)	0.108§
Ketamine + PCP + MDMA + Viagra + Nimetazepam + Others	1 (3.6)	0 (0.0)	1 (0.4)	0.108§

RUSH = alkyl nitrites or poppers, MDMA = 3,4-methylenedioxy-N-methylamphetamine or ecstasy, LSD = Lysergic acid diethylamide, PCP = Phencyclidine

*, Chi-square test.

^§^, Fisher exact test.

MSM using illegal drugs had a mean age of 30 years (range, 18–57 years), and most of them were single (93.1%), self-identified homosexuals (85.7%) and with educational levels ranging from college (62.9%) to graduate school (16.2%). Over half of the participants (58.7%) were office workers and 13.1% were students (Table 7). Univariate analysis showed that Ketamine, MDMA and Viagra use (vs. single drug use, OR = 6.46; p = 0.004) and use of oil-based lubricants during sexual intercourse (vs. saliva or water-based lubricants, OR = 4.18; p = 0.019) were significantly associated with HIV-1 infection (Table 8). Multivariate analysis demonstrated that the risk factors mentioned above remained significantly associated with HIV-1 infection in MSM who reported illegal drug use (Table 9). Furthermore, versatile role (vs. exclusively insertive role, OR = 6.38; p = 0.015) and Ketamine, MDMA and Nimetazepam use (vs. single drug use, OR = 60.12; p = 0.014) were also associated with HIV-1 infection in MSM using illegal drugs.

**Table 7:** Demographic data of patrons who reported illegal drug use from different gay venues participated in this study.

	HIV-1 (+)	HIV-1 (-)	Total	
Variable	N = 28	N = 231	N = 259	p
	n (%)	n (%)	n (%)	
Age				0.683§
18–29	12 (42.9)	99 (42.9)	111 (42.9)	
30–39	7 (25.0)	70 (30.3)	77 (29.7)	
40–49	1 (3.6)	16 (6.9)	17 (6.6)	
≥ 50	0 (0.0)	3 (1.3)	3 (1.2)	
NA	8 (28.6)	43 (18.6)	51 (19.7)	
Mean ± SD	28.3±5.0	30.2±7.4	30.0±7.2	0.255†
Marital status				0.216§
Single	24 (85.7)	217 (93.9)	241 (93.1)	
Married	1 (3.6)	5 (2.2)	6 (2.3)	
Divorced/Separated/Widowed	1 (3.6)	1 (0.4)	2 (0.8)	
NA	2 (7.1)	8 (3.5)	10 (3.9)	
Sexual orientation				0.666§
Heterosexual	0 (0.0)	5 (2.2)	5 (1.9)	
Homosexual	26 (92.9)	196 (84.9)	222 (85.7)	
Bisexual	2 (7.1)	28 (12.1)	30 (11.6)	
NA	0 (0.0)	2 (0.9)	2 (0.8)	
Education				0.381§
≤ Junior high school	1 (3.6)	5 (2.2)	6 (2.3)	
Senior high school	3 (10.7)	42 (18.2)	45 (17.4)	
College	22 (78.6)	141 (61.0)	163 (62.9)	
≥ Graduate	2 (7.1)	40 (17.3)	42 (16.2)	
NA	0 (0.0)	3 (1.3)	3 (1.2)	
Occupation				0.774§
Student	4 (14.3)	30 (12.9)	34 (13.1)	
Government employees	3 (10.7)	13 (5.6)	16 (6.2)	
Office worker	16 (57.1)	136 (58.9)	152 (58.7)	
Professional	1 (3.6)	18 (7.8)	19 (7.3)	
Unemployed/Other	4 (14.3)	28 (12.1)	32 (12.4)	
NA	0 (0.0)	6 (2.6)	6 (2.3)	
Place				0.637*
Gay bar (1)	8 (28.6)	57 (24.7)	65 (25.1)	
Gay night clubs (7)	15 (53.6)	144 (62.3)	159 (61.4)	
Gay saunas (3)	5 (17.9)	30 (12.9)	35 (13.5)	

*, Chi-square test.

^§^, Fisher exact test.

^†^, Student T Test.

**Table 8:** Univariate analysis of risk factors for HIV-1 infection among MSM who reported illegal drug use in Taiwan.

	HIV-1 (+)	HIV-1 (-)		
Variable	N = 28	N = 231	Odds ratio*	p
	n (%)	n (%)		
Pay for condom				
Yes	25 (89.3)	187 (80.9)	1	1
No	2 (7.1)	39 (16.9)	0.38	0.205
NA	1 (3.6)	5 (2.2)	1.49	0.718
Number of sexual partners				
0	2 (7.1)	20 (8.7)	1	1
1	13 (46.4)	90 (38.9)	1.44	0.645
2–3	7 (25.0)	67 (29.0)	1.05	0.959
≥ 4	0 (0.0)	21 (9.1)	<0.001	0.966
NA	6 (21.4)	33 (14.3)	1.82	0.489
Role during anal intercourse				
Exclusively insertive	3 (10.7)	60 (25.9)	1	1
Exclusively receptive	4 (14.3)	48 (20.8)	1.67	0.517
Versatile	19 (67.9)	111 (48.1)	3.42	0.055
Oral sex	1 (3.6)	1 (0.4)	20	0.051
NA	1 (3.6)	11 (4.8)	1.82	0.619
Times of sexual contact per week				
0	3 (10.7)	32 (13.9)	1	1
1	12 (42.9)	109 (47.2)	1.17	0.812
2–3	8 (28.6)	43 (18.6)	1.98	0.339
≥ 4	1 (3.6)	17 (7.4)	0.63	0.696
NA	4 (14.3)	30 (12.9)	1.42	0.662
Types or numbers of illegal drugs use				
Single drug	8 (28.6)	124 (53.7)	1	1
Ketamine + MDMA^a^	10 (35.7)	61 (26.4)	2.54	0.062
Ketamine + Viagra	1 (3.6)	11 (4.8)	1.41	0.757
Ketamine + Nimetazepam	0 (0.0)	1 (0.4)	<0.001	0.996
Ketamine + MDMA + Viagra	5 (17.9)	12 (5.2)	6.46	0.004
Ketamine + MDMA + Nimetazepam	1 (3.6)	1 (0.4)	15.5	0.061
Ketamine + MDMA + Viagra + Marijuana	0 (0.0)	7 (3.0)	<0.001	0.907
Ketamine + MDMA + Viagra + Nimetazepam	0 (0.0)	2 (0.9)	<0.001	0.950
Ketamine + MDMA + Marijuana + Nimetazepam	0 (0.0)	1 (0.4)	<0.001	0.965
Ketamine + MDMA + Viagra + Marijuana + Nimetazepam	1 (3.6)	3 (1.3)	5.17	0.175
Ketamine + PCP + MDMA + Viagra + Marijuana + Nimetazepam	1 (3.6)	0 (0.0)	>999.99	0.989
Ketamine + PCP + MDMA + Viagra + Nimetazepam + Others	1 (3.6)	0 (0.0)	>999.99	0.989
Others	0 (0.0)	8 (3.5)	<0.001	0.901
Sexual contact after illegal drug use				
Never	5 (17.9)	44 (19.1)	1	1
Rarely/Occasionally/ Frequently/Always	17 (60.7)	107 (46.3)	1.39	0.53
Condom use after illegal drug use				
Never	4 (14.3)	21 (9.1)	1	1
Rarely/Occasionally/ Frequently/Always	22 (78.6)	173 (74.9)	0.67	0.49
Oil- based lubricants during sexual intercourse				
Saliva or water- based	4 (14.3)	79 (34.2)	1	1
Oil- based^b^	11 (39.3)	52 (22.5)	4.18	0.019
NA	13 (46.4)	100 (43.3)	2.57	0.111
History of sexually transmitted diseases				
No	11 (39.3)	133 (57.6)	1	1
Yes	7 (25.0)	40 (17.3)	2.12	0.146
NA	10 (35.7)	58 (25.1)	2.09	0.114
Frequency of condom use				
Always	8 (28.6)	90 (38.9)	1	1
Frequently/Occasionally/ Rarely/Never	18 (64.3)	117 (50.7)	1.73	0.220
NA	2 (7.1)	24 (10.4)	0.94	0.938
Frequency of lubricant use				
Always	19 (67.9)	159 (68.8)	1	1
Frequently/Occasionally/ Rarely/Never	8 (28.6)	52 (22.5)	1.29	0.575
NA	1 (3.6)	20 (8.7)	0.42	0.408

*, Chi-square test;

^a^, MDMA = 3,4-methylenedioxy-N-methylamphetamine or so called ecstasy

^b^, Soap, baby oil, Vaseline and petroleum jelly

**Table 9:** Multivariate logistic regression of risk factors for HIV-1 infection among MSM who reported illegal drug use in Taiwan.

	HIV-1 (+)	HIV-1 (-)		
Variable	N = 28	N = 231	Odds ratio*	p
	n (%)	n (%)		
Role during anal intercourse				
Exclusively insertive	3 (10.7)	60 (25.9)	1	1
Exclusively receptive	4 (14.3)	48 (20.8)	1.75	0.557
Versatile	19 (67.9)	111 (48.1)	6.38	0.015
Oral sex	1 (3.6)	1 (0.4)	0.002	0.971
NA	1 (3.6)	11 (4.8)	5.12	0.224
Times of sexual contact per week				
0	3 (10.7)	32 (13.9)	1	1
1	12 (42.9)	109 (47.2)	1.38	0.709
2–3	8 (28.6)	43 (18.6)	2.55	0.297
≥ 4	1 (3.6)	17 (7.4)	1.49	0.766
NA	4 (14.3)	30 (12.9)	1.65	0.621
Oil-based lubricants during sexual intercourse				
Saliva or water- based	4 (14.3)	79 (34.2)	1	1
Oil-based ^a^	11 (39.3)	52 (22.5)	7.12	0.007
NA	13 (46.4)	100 (43.3)	3.06	0.113
History of sexually transmitted diseases				
No	11 (39.3)	133 (57.6)	1	1
Yes	7 (25.0)	40 (17.3)	2.59	0.121
NA	10 (35.7)	58 (25.1)	2.62	0.093
Types or numbers of illegal drugs use				
Single drug	8 (28.6)	124 (53.7)	1	1
Ketamine + MDMA^a^	10 (35.7)	61 (26.4)	2.62	0.079
Ketamine + Viagra	1 (3.6)	11 (4.8)	0.77	0.824
Ketamine + Nimetazepam	0 (0.0)	1 (0.4)	0.001	0.969
Ketamine + MDMA + Viagra	5 (17.9)	12 (5.2)	8.40	0.005
Ketamine + MDMA + Nimetazepam	1 (3.6)	1 (0.4)	60.12	0.014
Ketamine + MDMA + Viagra + Marijuana	0 (0.0)	7 (3.0)	<0.001	0.906
Ketamine + MDMA + Viagra + Nimetazepam	0 (0.0)	2 (0.9)	<0.001	0.944
Ketamine + MDMA + Marijuana + Nimetazepam	0 (0.0)	1 (0.4)	<0.001	0.965
Ketamine + MDMA + Viagra + Marijuana + Nimetazepam	1 (3.6)	3 (1.3)	5.16	0.209
Ketamine + PCP + MDMA + Viagra + Marijuana + Nimetazepam	1 (3.6)	0 (0.0)	>999.99	0.984
Ketamine + PCP + MDMA + Viagra + Nimetazepam + Others	1 (3.6)	0 (0.0)	>999.99	0.988
Others	0 (0.0)	8 (3.5)	<0.001	0.895

*, Chi-square test.

^a^, Soap, baby oil, Vaseline and petroleum jelly

## Discussion

In this study, we found that most HIV-1 seropositive MSM in Taiwan were infected with subtype B. In addition, phylogenetic analysis showed that they were clustered with local controls. Furthermore, we found that exclusively receptive, versatile roles and oral sex, 2–3 times of sexual contact per week and history of STDs, illegal drug use and use of oil-based solution as lubricants were important risk factors for HIV-1 infection among MSM in Taiwan.

The present study reported a 4.38% prevalence of HIV-1 in MSM in Taiwan in 2012. This figure differs from that of a similarly published report that indicated a variable but considerably higher prevalence of HIV-1 among MSM visiting gay saunas in Taiwan between 2001 and 2005 [14]. According to results of syphilis testing, 2.15% of participants in this study were infected with syphilis. Syphilis infections significantly increase the risk of HIV infection. Some studies have reported that after controlling for other risk factors, participants who had been infected with syphilis were more likely to be infected with HIV [31, 32].

The BED-CEIA is the most commonly used assay for estimating HIV-1 incidence [33]. However, several previous studies in different populations with different HIV-1 subtype infections showed that LAg-Avidity EIA had a lower false recent rate and higher accuracy than BED-CEIA [34–36]. Therefore, we used LAg-Avidity EIA in our analysis. The results showed that 16 of 53 HIV-1 infected MSM were recent seroconverters and the resultant incidence was 3.29 per 100 person-years. False recent infection in HIV-1 seropositive patients can be due to antiretroviral therapy (ARV), elite controllers, terminal stage of AIDS, and non-B subtype infection (WHO/UNAIDS Technical Update on HIV incidence assays for surveillance and epidemic monitoring, 2013). However, none of the factors mentioned above are present in our study: 15 of 16 (93.8%) recent seroconverters detected in our study were treatment naïve patients with subtype B infection and the remaining one (6.3%) was a treatment naïve patient with CRF01_AE infection.

In this study, we found that 97.9% of all infections were caused by subtype B. This was not surprising given that previous reports had already established B as the most predominant subtype among Taiwanese MSM [14, 37, 38]. Of the non-subtype B infections, 2% was attributed to CRF01_AE. Although CRF01_AE is predominantly found among female heterosexuals, several studies have speculated on their possible transmission to other risk groups [5, 7, 39]. The results of HIV-1 subtyping were consistent between gag subtype-specific PCR (48 patients infected with subtype B and 1 patient infected with CRF01_AE) and phylogenetic analysis (45 patients infected with subtype B and 1 patient infected with CRF01_AE). The rationale for using both gag subtype-specific PCR and DNA sequencing of env for genotyping was because the former method can detect dual or even triple-subtype infection and the latter can be used for phylogenetic tree analysis [7, 39].

Phylogenetic analysis of viral gene sequences has successfully been used to construct direct or indirect epidemiological links in geographically defined populations with acute/primary or chronic HIV-1 infection [40, 41]. The epidemic of HIV-1 infection in MSM has expanded rapidly in Asian countries [42, 43]. Our previous study showed that CRF07_BC was transmitted into Taiwan from China and subsequently caused an epidemic in the Taiwanese IDU population [3, 5]. A previous study showed that CRF07_BC infection has spread to the heterosexual population [7]. In this study, 97.9% MSM were infected with subtype B and CRF07_BC did not transmit into this population. A recent study demonstrated that CRF07_BC had significantly lower viral loads than subtype B [44]. This may explain why CRF07_BC cannot transmit through sexual contact as efficiently as through needle sharing.

In this study, 13 clusters were identified in the phylogenetic analysis of 46 env sequences. In all subtype B clusters, MSM sequences aggregated among themselves or with the local control sequences. The CRF01_AE strain (G249) clustered with a reference sequence from Thailand with a bootstrap value of 99%. This sequence was from a Thai blood donor [45]. No further details about the Thai individual could be obtained to better assess the association between these two sequences. However, G249 claimed that he had joined a sex tour in Thailand. Since CRF01_AE is predominant among MSM in China and Thailand [46–48], further surveillance of the prevalence of CRF01_AE among MSM in Taiwan is needed to monitor its transmission dynamics in this population.

Compared to heterosexual males, MSM are more susceptible to HIV-1 infection. Cultural, social, political and religious factors that lead to denial of MSM and their sexual practices increase their vulnerability [49]. These are important aspects that HIV prevention programs should take into account. MSM are also known to face additional challenges that may lead to sexual risk taking behavior and increase their chances for HIV seroconversion. This has been well-documented throughout the epidemic and confirmed in this study. Previous studies demonstrated that receptive and versatile roles during anal intercourse were associated with an increased risk of HIV seroconversion among MSM [32, 50]. Generally, the receptive partner was at greater risk of contracting HIV because the lining of the rectum is thin and may allow the virus to enter the body through semen exchange [32, 51]. In a previous study among MSM visiting gay saunas in Taiwan between 2001 and 2005, men assuming receptive and versatile roles during anal intercourse were also identified to be at higher risk of seroconversion [14].

Illegal drug use among MSM is one of the most cited risk factors associated with HIV-1 infection. In this study, the odds of HIV-1 seroconversion in MSM who used illegal drugs was 4.12 times higher than in those who did not use illegal drugs (OR = 4.12; P <0.001). The relationships between substance use, high-risk sexual behaviors and HIV infection are complex. It is believed that illegal drug use among MSM may be either a trigger or an excuse for engaging in unprotected sex [52, 53].

In this study, compared to illegal use of a single drug, alternate use of Ketamine and MDMA and either Viagra or Nimetazepam was associated with HIV-1 infection. Ketamine has powerful hallucinogenic qualities and makes the user feel disassociated from his or her body. The user may feel sleepy or sluggish, or confused and clumsy [54]. MDMA is a synthetic hallucinogenic stimulant. The immediate effect of MDMA is a feeling of euphoria. It makes the user feel more comfortable in social situations [55]. Viagra is an orally administered prescription medication to treat erectile dysfunction. Nimetazepam possesses hypnotic, anxiolytic, sedative, and skeletal muscle relaxant properties. MDMA is usually not taken with Ketamine and Nimetazepam due to the different effects of the drugs. Several studies has shown that use of two or more psychoactive drugs (polydrug use) places individuals at greater risk of engaging in unprotected sex and thus increases the risk of HIV seroconversion [56–58]. However, participation in risky sexual behavior and risk of HIV seroconversion vary greatly by type of substances used. Polydrug use may increase the risk of HIV seroconversion since it can interfere with the adherence or the effectiveness of antiretroviral therapy [12, 59]. Therefore, having unprotected anal intercourse with an HIV-infected partner who frequently engages in polydrug use increases the probability of HIV acquisition as these persons may be highly infectious due to a reduction in the effectiveness of their treatment. Moreover, ingesting a combination of drugs may lead to transient immune suppression [60]. Several studies have also identified reasons for polydrug consumption among MSM, including the potentiating effect associated with mixing drugs [61].

Participants with a history of STDs were also more likely to be infected with HIV (OR = 3.65; p = 0.002). Research suggests that STDs can increase both an HIV-negative person’s risk of becoming infected with HIV and an HIV-positive person’s risk of transmitting HIV to someone else [50, 62]. Having an STD increases a person’s risk of becoming infected with HIV due to various biological mechanisms. STDs increase the concentration of dendritic cells and CD4 T-cells in the genito-anal region, which is the most usual site of STDs infection [63]. The presence of an STD in a co-infected person increases the chances of transmitting HIV through open genital lesions, which come into contact with the partner’s anal and oral mucous membranes during sexual activity [31].

The inappropriate use of lubricants among MSM is not a new phenomenon, as it was observed in high-income settings nearly two decades ago [64]. Surprisingly, we found a very high percentage of oil- or petroleum-based solution misuse as lubricants in this study (47.2% in HIV-1-infected MSM vs. 25.9% HIV-1 sero-negative MSM). Oil-based lubricants misused included Vaseline (14%), baby oil (6%), soap (4%) and other types of lotion (6%). Multivariate analysis showed that participants using such lubricants were more likely to be infected with HIV than those using saliva or water-based lubricants (OR = 4.23; p <0.001). In addition, data analysis combined with in-depth interview at post-test counseling showed that among 53 HIV-1 seroconverters, 12 (22.6%) MSM used condoms consistently but misused oil-based solution as lubricants. Since most of them played a receptive role (bottom) during anal intercourse, they were not aware of the breakage of condoms during or after sexual intercourse. This is a very important risk factor that has been neglected in AIDS education for a long time. Similar phenomena may exist among MSM in other countries and this should be taught in AIDS education campaign. The Taiwan’s Centers for Disease Control has produced a video to correct this misconception (www.youtube.com/watch?v=BinExvvOTMM&feature=iv&src_vid=BW81-PfmY3E&annotation_id=annotation_2436493705). Our previous study showed that uncircumcised, versatile role during anal intercourse, and having sex with more than one person during each sauna visit were main risk factors for HIV-1 infection in Taiwan from 2001 to 2005 [14]. A previous study by Ko et al showed that illegal drug use was the primary risk factor for HIV infection [12]. A study in China showed that the number of sex partners and frequency of anal sex were associated with HIV-1 infection among MSM [15]. A cohort study conducted between April 2006 and July 2012 in Thai MSM showed that drug use for sexual pleasure and receptive anal intercourse were associated with HIV-1 infection [65]. Risk factors that are consistently present in different studies were receptive role during anal intercourse and illegal drug use. Misuse of oil-based solutions as lubricant and illegal drug use are risk factors that have never being mentioned previously.

Overall, 4.38% of MSM participating in this study were infected with HIV. Among the infected participants, the majority were infected with subtype B. Multivariate analysis showed that risk factors of HIV-1 seroconversion among MSM included an exclusively receptive role during anal intercourse, multiple sexual contacts per week, use of illegal drugs, use of oil-based lubricants during sexual intercourse and history of STDs.

The burden of HIV among MSM in Taiwan is disproportionately high. The results of this study can be useful for the design and implementation of evidence-based interventions aimed at preventing and controlling HIV and STDs among MSM, supporting safe sexual behavior, promoting uptake of HIV testing, and promoting HIV and STD health care seeking behavior.

## Supporting Information

S1 Fig Maximum likelihood tree analysis based on Env nucleotide sequence of MSM in Taiwan in 2012. (TIF)

S1 Table Characteristics of the clusters in phylogenetic tree analysis. (DOC)
