# Supplementary figures and images for: Molecular Epidemiology of HIV-1 Infection among Men who Have Sex with Men in Taiwan in 2012
Source: PLoS One. 2015 Jun 3;10(6):e0128266. doi: 10.1371/journal.pone.0128266 (PMC4454672; doi:10.1371/journal.pone.0128266)

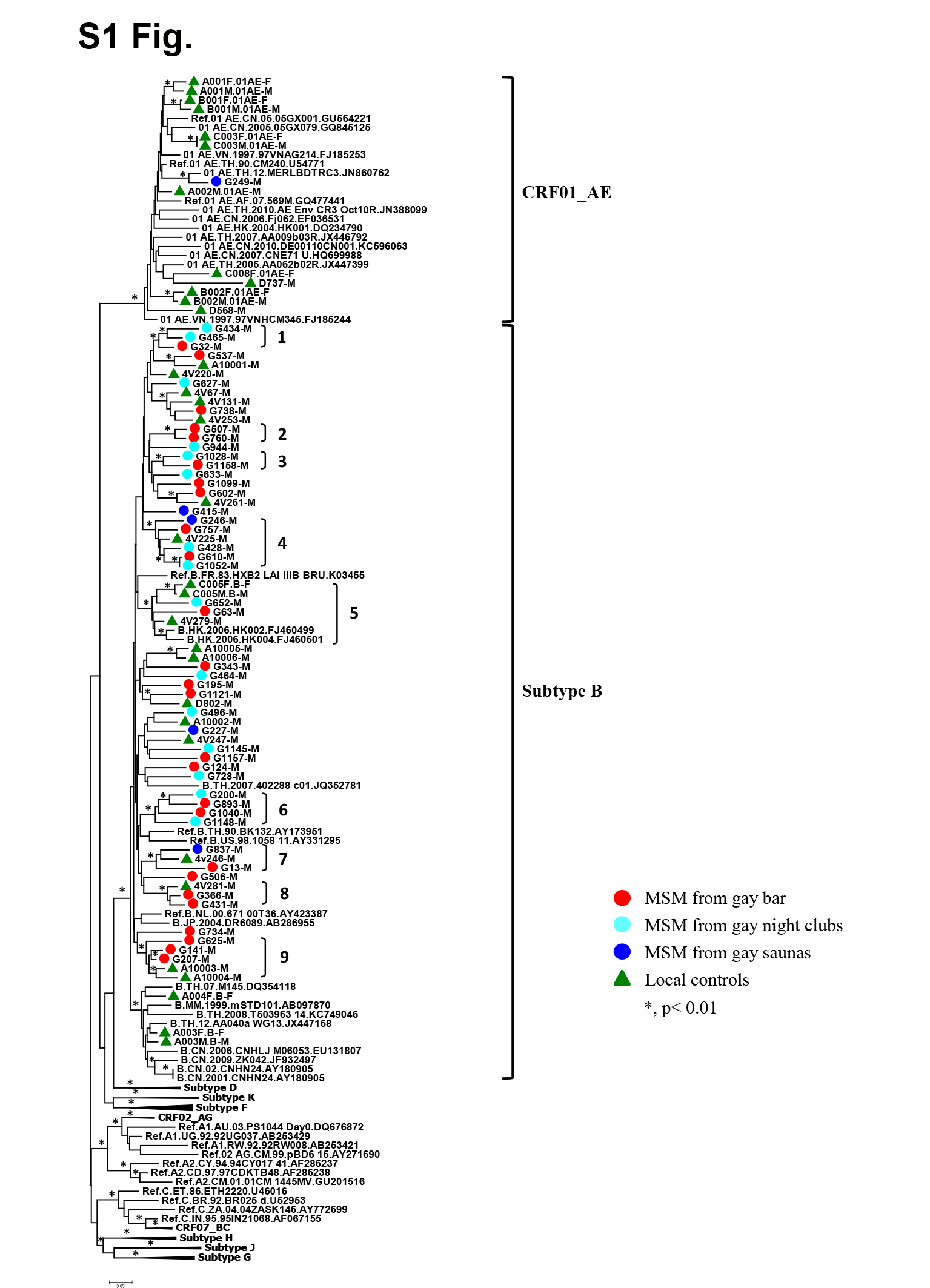

Supplement: S1 Fig — (TIF) [file pone.0128266.s001.tif]
