# Supplementary material for: Molecular Epidemiology of HIV-1 Infection among Men who Have Sex with Men in Taiwan in 2012
Source: PLoS One. 2015 Jun 3;10(6):e0128266. doi: 10.1371/journal.pone.0128266 (PMC4454672; doi:10.1371/journal.pone.0128266)
Supplement: S1 Table — (DOC) [file pone.0128266.s002.doc]

| **Variable** | **Cluster number** | | | | | | | | | **Total** |
| --- | --- | --- | --- | --- | --- | --- | --- | --- | --- | --- |
|  | **1** | **2** | **3** | **4** | **5** | **6** | **7** | **8** | **9** |  |
| Number of patrons | 3 | 2 | 2 | 5 | 2 | 4 | 2 | 2 | 3 | 25 |
| Age (years) |  |  |  |  |  |  |  |  |  |  |
| Young (Y) | 🗸 |  | 🗸 |  | 🗸 |  |  | 🗸 |  | 4 |
| Mix (M) |  |  |  | 🗸 |  |  | 🗸 |  |  | 2 |
| NA |  | 🗸 |  |  |  | 🗸 |  |  | 🗸 | 3 |
| Illegal drug use |  |  |  |  |  |  |  |  |  |  |
| All of them (All) |  |  | 🗸 |  |  |  |  |  |  | 1 |
| None of them (None) |  |  |  |  |  |  |  | 🗸 |  | 1 |
| Some of them (Some) | 🗸 | 🗸 |  | 🗸 | 🗸 | 🗸 |  |  | 🗸 | 6 |
| NA |  |  |  |  |  |  | 🗸 |  |  | 1 |
| Characteristics  (Age + illegal drug use) | Y | NA | Y | M | Y | NA | M | Y | NA |  |
|  | + | + | + | + | + | + | + | + | + |  |
|  | Some | Some | All | Some | Some | Some | NA | None | Some |  |

**S1 Table. Characteristics of the clusters in phylogenetic tree analysis.**

Cluster was defined as bootstrap value ≥70 using phylogenetic analysis.

NA, data not available
